# A new ureteroileal anastomosis technique in modified ileal orthotopic bladder substitution after radical cystectomy

**DOI:** 10.1186/s12957-020-01831-w

**Published:** 2020-04-11

**Authors:** Bin Wu, Chunyu Pan, Zichuan Yao, Xianqing Zhu, Song Bai

**Affiliations:** Department of Urology, Shengjing Hospital of China Medical University, 36 Sanhao Street, Shenyang, 110004 Liaoning People’s Republic of China

**Keywords:** Bladder cancer, Reflux, Ureteroileal anastomosis, Hydronephrosis, Orthotopic bladder substitution

## Abstract

**Background:**

The aim of this study is to describe a modified technique in ileal orthotopic bladder substitution with a new ureteroileal anastomosis.

**Case presentation:**

After a classic open radical cystectomy with bilateral pelvic lymphadenectomy was performed extraperitoneally, a 56-cm ileal segment was used to construct the spheroidal shape bladder substitution. The 8-cm long proximal and distal ends of the ileal segment were used as afferent limbs. Two-centimeter ileal segments of afferent limbs were detubularized and transversally tubularized. The elongated ileal tube was anastomosed with the ureter in an end-to-end fashion. The pathway of the ureteroileal anastomosis was placed between the abdominal cutaneous fat and the anterior rectus muscular sheath. Perioperative data and long-term functional outcomes were assessed. Between December 2011 and December 2015, seven male patients underwent this procedure with a median 46 (30–77) months follow-up in our hospital. There was no difference between preoperative and postoperative estimated glomerular filtration rates (*Z* = − 1.693, *P* = 0.09). One of 14 sides had ureteroileal anastomotic stenosis; two of 14 sides in one patient had ureteroileal anastomotic stenosis caused by invasion of pelvic recurrence 15 months postoperatively. Reflux was completely prevented by placing pressure on the corresponding point on the abdominal surface when voiding urine in all patients.

**Conclusions:**

We describe a feasible technique modification in detail, which provides some advantages for effective anti-reflux by mechanical finger pressing and abdominal contraction, a low incidence of stricture, and ease for a secondary operation in the long-term follow-up period.

## Background

Although radical cystectomy coupled with pelvic lymphadenectomy remains the standard surgical approach for muscle-invasive bladder cancer, the optimal mode of urinary tract reconstruction following radical cystectomy remains challenging. Ileal orthotopic bladder substitution is one of the most popular techniques, and could provide unchanged voiding habits [1, 2]. The satisfaction and quality of life associated with it are incomparable to other types of urinary diversion in younger patients [3].

Preservation of the morphology and function of the upper urinary tract are extremely important in ileal orthotopic bladder substitution. However, complications of ureteroileal anastomosis are very common and difficult to manage, particularly ureteroileal anastomosis stenosis and vesicoureteral reflux [4]. The types of ureteroileal anastomosis are divided into anti-reflux and reflux techniques. Neither of these is perfect. The literature has implied that anti-reflux anastomotic techniques are effective in preventing reflux but have more stenotic complications [5, 6]. Reflux techniques are relatively easier to perform with lower stenosis rates in long-term follow-up, but also might have drawbacks on renal function because of vesicoureteral reflux, especially during voiding due to increasing bladder luminal pressure [7].

Hence, we sought to identify a new technique that would resolve these problems by implementing a new method of ureteroileal anastomosis in an ileal-modified orthotopic bladder substitution. Outcomes and complications were evaluated through an extended follow-up period.

## Case presentation

### Patients

We retrospectively evaluated the records of seven patients who underwent modified ileal orthotopic bladder substitution after radical cystectomy in our hospital between December 2011 and December 2015. Ethical approval (Ethical Committee no. 2018PS399K) was provided by the Institutional Research and Ethics Committee of the Shengjing Hospital Affiliated China Medical University in Shengyang, China. Informed consent from all eligible patients was obtained and we emphasized in very informed consent that part of this surgery would adopt a new procedure. Registration UIN is ChiCTR1800018185.

Inclusion criteria were as follows: cT2-4a N0/1 M0 stage without urethral and bladder neck invasion, muscle invasive bladder cancer, and American Society of Anesthesiologists (ASA) score of 1–2, male patients. Exclusion criteria were as follows: BMI > 30, estimated glomerular filtration rate (eGFR) < 60 ml/min as measured by the Modification of Diet in Renal Disease formula, chronic intestinal disease or previous abdominal surgery, pelvic radiation, impaired dexterity, and decreased intellectual capacity. Patient characteristics were assessed. The bladder cancer was classified according to the 2017 TNM classification [8]. Histological grading was made according to the 2004 WHO grading system (https://www.ncbi.nlm.nih.gov/pubmed/9850170).

Perioperative data were assessed. Complications were graded according to the Clavien system [9], and 3 months after surgery was the boundary between short-term and long-term complications. All procedures were performed by a single surgeon with advanced surgical skills. This study was in line with the PROCESS criteria [10].

### Preoperative counseling and preparation

A detailed discussion about potential complications of radical cystectomy, as well as the advantages and disadvantages of each method of urinary diversion was performed with the patients preoperatively. Standard mechanical and oral antibiotic bowel preparation was initiated 3 days prior to surgery in each patient.

### Surgical techniques

Patients underwent surgery under general anesthesia in the Trendelenburg position. A standard lower midline abdominal incision was made. A classic radical cystectomy was performed, with excision of the prostate, seminal vesicles, and ampulla of the vas deferens extraperitoneally with preservation of the urethral sphincter. Bilateral pelvic lymphadenectomy was performed; the lymph nodes around the common, external, and internal iliac vessels, as well as the obturator vessels, were removed. The distal ureters on both sides were dissected and sectioned close to the bladder as low as possible.

**Fig. 1:**
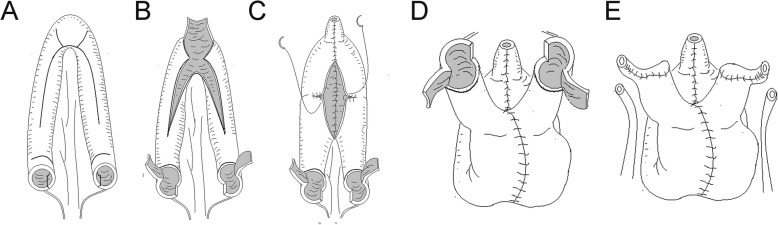
Construction of a modified ileal orthotopic bladder substitution. **a** Excision of a 56-cm ileal segment at least 25 cm proximal to the ileocecal junction was placed as a U shape. A 40-cm intermediate part was opened along the anti-mesenteric border. **b** A 5-mm diameter enterotomy was made at the apex of the U shape, where it was opened towards the mesenteric border and ready for anastomosis with the urethra. The medial borders of the incision were closed in a longitudinal fashion with 3-0 PDS continuous sutures to form the posterior wall of the neobladder. **c** and **d** The intermediate 40-cm ileal segment was used to construct the spheroidal-shaped neobladder. The 8-cm long proximal and distal ends of the ileal segment were used as afferent limbs, which were ready to anastomose with the ureter. **e** The elongated ileal tube was formed and ready to anastomose to the ureters

**Fig. 2:**
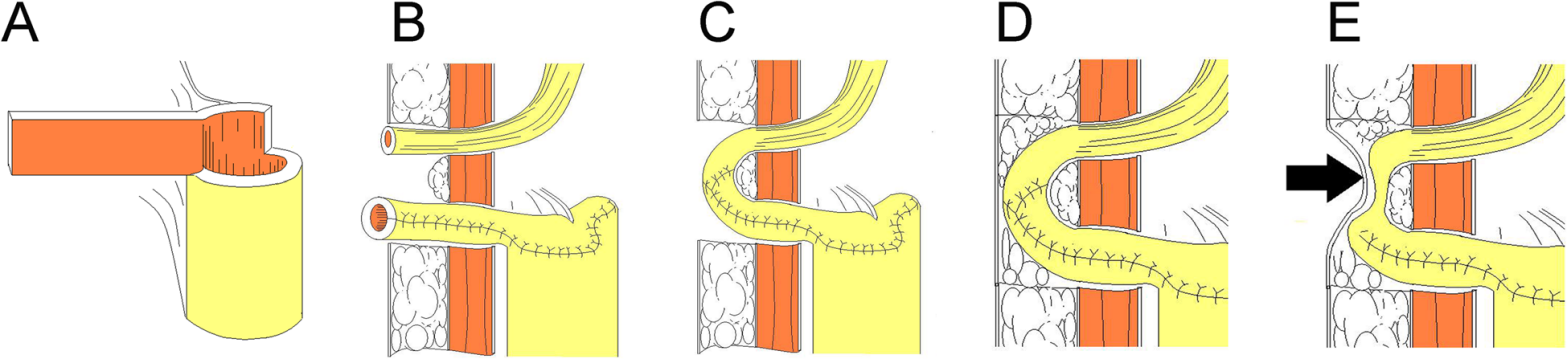
Mode chart: construction of elongated ileal tube and anastomose with ureter. **a** A 2-cm ileal segment was detubularized and then transversally tubularized. The elongated ileal tube was formed and ready to anastomose with the ureter. **b**, **c**, and **d** The ureter and elongated ileal tubes were anastomosed in an end-to-end fashion. The pathway of the ureteroileal anastomosis was placed between the abdominal cutaneous fat and the anterior rectus muscle sheath. **e** Reflux could be completely prevented by placing pressure on the corresponding point on the abdominal surface when straining for voiding urine

**Fig. 3:**
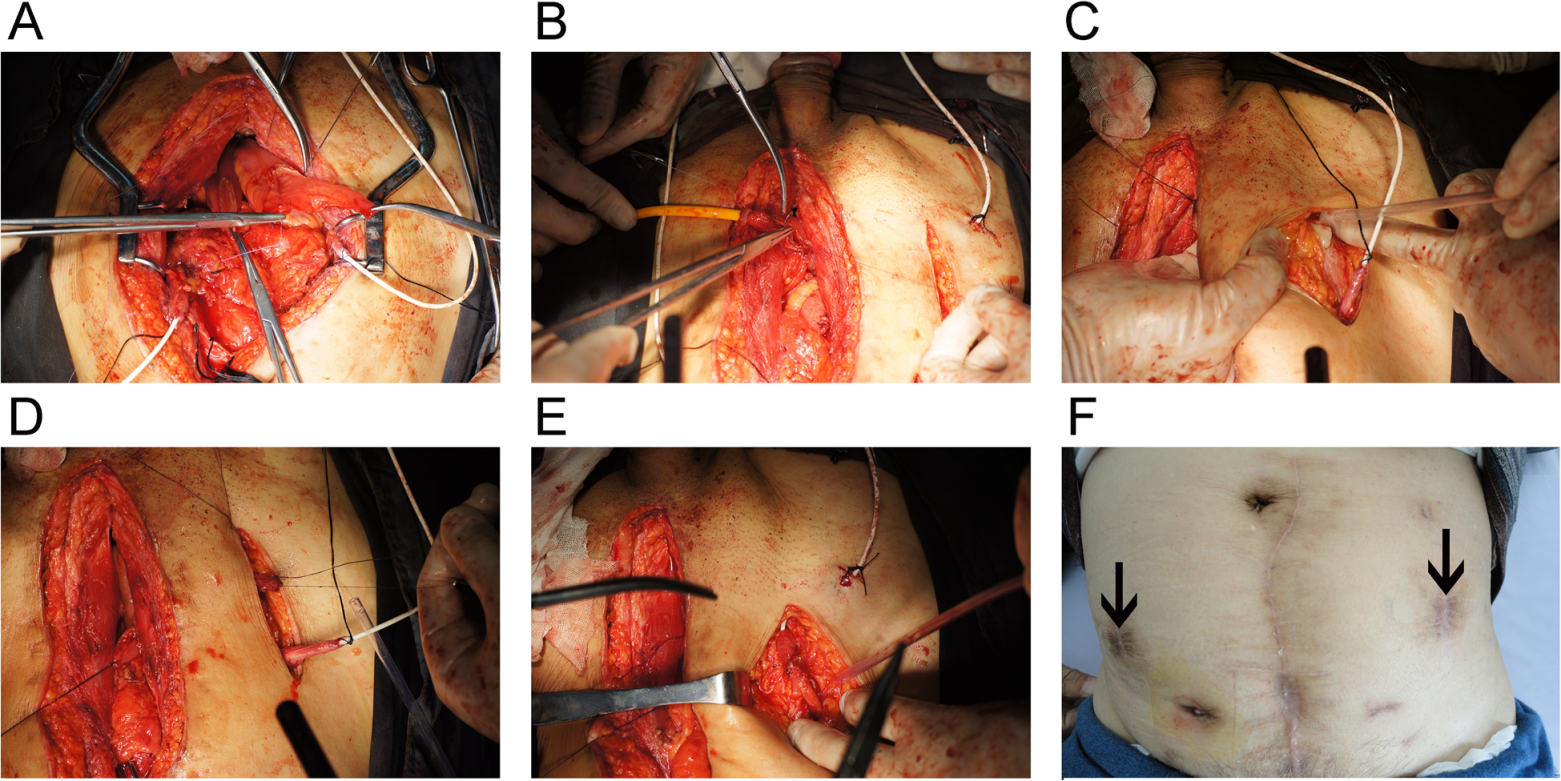
Operation chart construction of elongated ileal tube and anastomosis with ureter (right side). **a** and **b** The elongated ileal tube was formed. **c, d**, and **e** The ureter and elongated ileal tubes were anastomosed in an end-to-end fashion. The pathway of the ureteroileal anastomosis was placed between the abdominal cutaneous fat and the anterior rectus muscular sheath. **f** Reflux could be completely prevented by pressing the corresponding point on the abdominal surface when straining for voiding urine. The corresponding abdominal surface position was McBurney’s point and anti-McBurney’s point. The solid arrow indicates the pressure point


We opened the peritoneum and harvested a 56-cm ileal segment at least 25 cm proximal to the ileocecal junction and restored ileum continuity later; it was placed in a U shape. A 40-cm section in the intermediate portion was opened along the antimesenteric border. A 5-mm diameter enterotomy was made at the apex of the U shape, where it was opened towards the mesenteric border and ready for anastomosis with the urethra. The medial borders of the incision were closed in a longitudinal fashion with 3-0 PDS continuous sutures, to form the posterior wall of the neobladder. The intermediate 40-cm ileal segment was used to construct the spheroidal-shaped neobladder, as shown in Fig. 1. The 8-cm long proximal and distal ends of the ileal segment were used as afferent limbs, ready to anastomose with the ureter. A 2-cm ileal segment was detubularized through the longitudinal incision, halfway on the lateral side of the afferent limb in the modified ileal orthotopic bladder substitution. It was then transversally tubularized with interrupted 4-0 PDS sutures according to the Monti principle [11]. The elongated ileal tube was formed and ready to anastomose with the ureter. The flap was trapezoidal with a base top ratio of 2:1 that could provide sufficient blood supply. The ureter and elongated ileal tubes were anastomosed with interrupted PDS 4-0 sutures in an end-to-end fashion. The ureters were stented with a seven-French single J stent, brought out from the anterior wall of neobladder, and anchored to the skin. The pathway of the ureteroileal anastomosis was unusually placed between the abdominal cutaneous fat and the anterior rectus muscular sheath, instead of the intraperitoneal cavity. Some fat was left beneath the anastomosis to avoid the formation of an acute angle that could easily lead to stenosis. The corresponding abdominal surface position was McBurney’s point and anti-Mcburney which need longitudinal 5-cm incision for this anastomosis (Figs. 2 and 3, video 1).

**Additional file 1: Video 1.** A new ureteroileal anastomosis technique in modified ileal orthotopic bladder substitution after radical cystectomy.


Finally, the ileal orthotopic bladder substitution was anastomosed to the urethra with eight interrupted 2-0 PDS sutures. A 22-French silicon Foley catheter was placed in the urethra and a bladder fistula drain was placed in the neobladder before completely closing the pouch. Then, the neobladder dome was suspended to the back of the rectus abdominus muscle and the peritoneum was closed. The pelvis was drained with a 28-French tube drain. We checked for leakage after the neobladder was constructed.

**Fig. 4:**
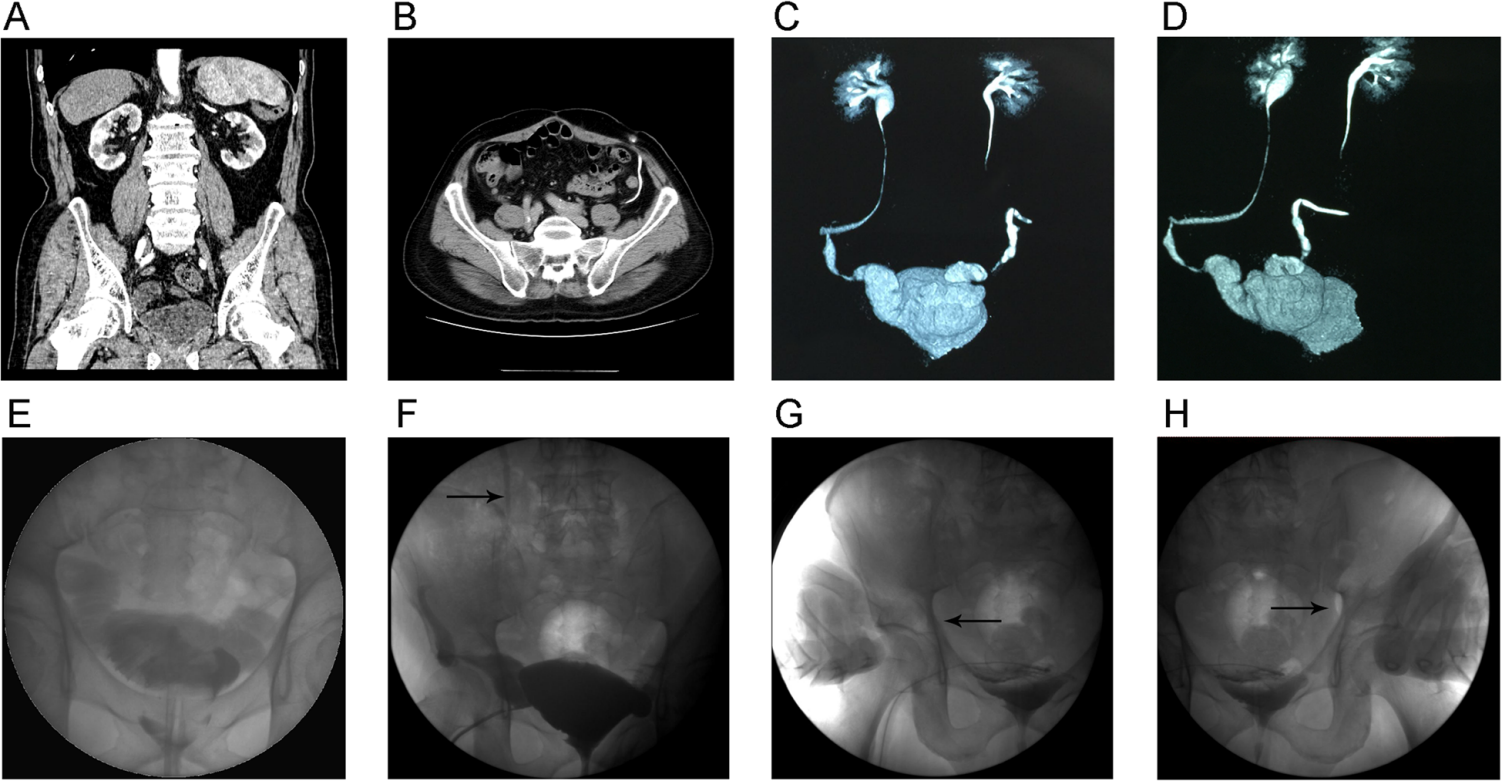
Computed tomography scan of urinary system and cystography data after the operation. **a** Coronal position of CT. **b** Horizontal position of CT. **c** Three-dimensional (3D) enhanced CT (anterior-posterior position). **d** 3D enhanced CT (lateral position). **e** Bladder capacity of cystography. **f** Right side: vesicoureteral reflux during urination of cystography, solid arrow indicating vesicoureteral reflux. **g** and **h** Right side and left side: vesicoureteral reflux could be completely prevented by pressing the corresponding point on the abdominal surface when straining for voiding urine; solid arrow indicates the pressure point

The ureteral stents were removed during the third postoperative week. The urethral Foley catheter was removed during the fourth week, then the bladder fistula drain was removed the following day. Patients were taught perineal exercises and pelvic relaxation techniques. They were instructed to void first by relaxing the pelvic floor, and then by abdominal straining to achieve complete bladder emptying, while pressing the body surface of the corresponding ureteroileal anastomosis with both thumbs to prevent reflux (Fig. 4).

### Follow-up

All patients were evaluated postoperatively at 3-month intervals for the first year and at 6-month intervals for the second year. Follow-up examinations involved routine blood and urine chemistry studies, renal ultrasonography, chest and abdominal radiography, cystography, and cystoscopy. We focused on stenosis of the ureteroileal anastomosis, vesicoureteral reflux, and renal function. Data about bladder capacity, urinary continence, urinary residual, and urinary tract infection were also collected, the follow-up period was calculated from the date of surgery to the date of death, or the final visit with the patient.

### Statistical methods

Continuous variables with non-normal variables were reported as the median (interquartile range). Categorical data are presented as frequency and percentages. The mean of two continuous non-normally distributed variables were compared by the Wilcoxon signed-rank test. Statistical analyses were performed using SPSS 22.0 or Windows (SPSS Inc., Chicago, IL, USA). A *P* value of less than 0.05 was considered statistically significant.

## Results

There were seven patients who underwent this operation with long-term follow-up (median 46 months, range 30–77 months). The median age and body mass index (BMI) were 64 years and 25.1 kg/m^2^ respectively. All patients were male whose bladder tumors were high grade and muscle invasive stages. One patient had pelvic lymph node metastasis (Table 1).

**Table 1:** Baseline characteristic data of the cohort

Characteristics	Results
No. of cases	7
Sex, male/female	7 (100)/0
Age (years), median (IQRs)	64 (60–73)
BMI (kg/m^2^), median (IQRs)	25.1 (24.3–26.3)
Tumor grade, high/low	7 (100)/0
Clinical stage	
T2N0M0	1 (14.3)
T3N0M0	5 (71.4)
T3N1M0	1 (14.3)

*No.* number, *BMI* body mass index, *IQR* interquartile range, data presented as median (IQRs) or no. (percentages)

Median operative time was 360 min, median estimated blood loss was 600 ml, four of seven patients underwent blood transfusion, and the median postoperative stay was 21 days, six of seven patients had short-term complications in which two of seven had non-compressive lymphoceles, and three of seven had urinary retention caused by a mucous plug, requiring 5 days of indwelling catheterization and irrigation. One patient had an asthmatic exacerbation and recovered within 5 days after therapy in the intensive care unit.

Preoperative and postoperative eGFR were 76 ml/min and 72 ml/min, respectively; there was no statistically significant difference according to the Wilcoxon signed-rank test (*Z* = − 1.693, *P* = 0.09). Two of seven patients and three of 14 sides had long-term complications, including a left ureteroileal anastomotic stenosis in a 75-year-old patient who subsequently underwent unilateral ureterocutaneoplasty under local anesthesia 6 months postoperatively. One patient who had metastasis in one lymph node developed pelvic recurrence of the tumor, which invaded the ureteroileal anastomosis and led to bilateral hydronephrosis 15 months postoperatively; this patient also underwent bilateral ureterocutaneoplasty under local anesthesia 25 months after surgery (Table 2). The other patients had no hydronephrosis after long-term follow-up (Fig. 4). Reflux was completely prevented by placing pressure on the corresponding point on the abdominal surface while straining for voiding urine in all patients (Fig. 4). Median neobladder capacity was 550 ml, median residual urine was 20 ml, and median neobladder pressure at maximal capacity was 20 cm H_2_O. None of the patients developed urinary incontinence, urinary tract infection, urethral stenosis, or metabolic acidosis.

**Table 2:** Perioperative data of the cohort

Characteristics	Results (***n*** = 7)
Follow-up time (months), median (range)	46 (30–77)
Operative time (min), median (IQRs)	360 (350–450)
Estimated blood loss (ml), median (IQRs)	600 (500–800)
Patients receiving transfusion (no./percent)	4 (57.1)
Postoperative hospital stay (day), median (IQRs)	21 (21–26)
**Short-term perioperative complication** (**< 3 months**),*n (**%**)	
Clavien I non-compressive lymphocele	2 (28.6)
Clavien I urine retention by mucous plug	3 (42.9)
Clavien IVa asthma attack	1 (14.3)
**Long-term perioperative complication** (**> 3 months**),*n (**% sides**)	
Clavien IIIb ureteroileal anastomosis stenosis (left vs. right)	2 (14.2)/1 (7.1)
eGFR (ml/min), median (IQR) (preoperative vs. postoperative)	76 (70–77)/72 (67–80)#
Intestinal obstruction	0 (0)
Urinary incontinence (no./percent)	0 (0)
Urinary retention (no./percent)	3 (42.9)
Urethral stenosis	0 (0)
Urinary tract infection	0 (0)
Metabolism acidosis	0 (0)
Neobladder capacity (ml), median (IQRs)	550 (500–650)
Residual urine (ml), median (IQRs)	20 (15–30)
Neobladder pressure at maximum capacity (cm H_2_O), median (IQRs)	20 (10–25)
Local recurrence	1 (14.3)

*eGFR* estimated glomerular filtration rate, *IQR* interquartile range, data presented as median (IQRs) or no. (percentages)

*According to Clavien classification of surgical complications.

#Wilcoxon signed-rank test, *Z* = − 1.693, *P* = 0.09

## Discussion and conclusions

When constructing orthotopic bladder substitution, a design with features similar to that of a normal bladder must be adopted, including creating a low pressure pouch with adequate capacity, effective preservation of renal function [12]. Ileal orthotopic bladder substitution, which is the most popular technique, provides unchanged voiding habits [12]. However, controversies remain regarding the optimal mode of ureteroileal anastomosis. Anti-reflux techniques such as the intussuscepted nipple valve, direct submucosal ureteral implantation, and the Le Duc technique, can be harmful to renal function due to the development of anastomotic strictures at a higher rate than with refluxing techniques (9–20% vs. 1–6%) [13]. A randomized trial of 60 patients undergoing the anti-reflux technique also demonstrated higher stricture (10%) and renal dysfunction rates compared with reflux anastomosis [14]. Refluxing techniques, such as the Nesbit technique, are easier to perform with a lower stenosis rate in the long-term follow-up period; but these techniques also have drawbacks for renal function, including recurrent pyelonephritis and hydronephrosis caused by vesicoureteral reflux, especially during voiding due to increasing bladder luminal pressure. Song et al. [7] reported that of 73.2% (101/138) patients who have vesicoureteral reflux, 80.0% were bilateral and 31.7% were more than level III reflux, which would impair renal function. Hence, we designed a new technique that would resolve these problems by using new methods of ureteroileal anastomosis in an ileal-modified orthotopic bladder substitution.

In this study, all patients were male with good physical status (ASA score of 1–2). The BMI of patients were less than 30 kg/m^2^ as such ureteroileal anastomosis can be more easily disturbed upon more adipose tissue. All patients had high grade, muscle invasive urothelial carcinoma, and only one had pelvic lymph node metastasis, age 38, with a strong desire for orthotopic bladder substitution.

The fate of the upper urinary tract depends on a combination of factors, including ureteroileal obstruction, and vesicoureteral reflux [5]. In this study, renal function can be preserved effectively, over a long-term follow-up period (range 30–77 months). There was no statistically significant difference between preoperative and postoperative eGFR (*P* = 0.09), and only two of seven patients and three of 14 sides had ureteroileal anastomotic stenosis. Of these, only one side occurred in ureteroileal anastomotic stenosis 6 months postoperatively; the other two sides of stenosis were caused by pelvic tumor recurrence invasion in one patient. Both patients received ureterocutaneoplasty under local anesthesia successfully.

There are still controversies in the optimal mode of ureteroileal anastomoses. Anti-refluxing techniques such as Le Duc technique, the serous-lined extramural tunnel, the intussuscepted niple valve, and the split-cuff ureteric nipple have harmful effect on renal function caused by anastomotic strictures which are higher than refluxing technique (9–20% vs. 1–6%) [15, 16]. Refluxing techniques have also drawback on renal function of recurrent pyelonephritis caused by reflux especially during voiding due to increasing bladder luminal pressure. However, it should be noted that reflux-associated complications with orthotopic neobladders have been predominantly seen in patients with high pressure reservoirs; contemporary neobladder designs use detubularized bowel segments configured to provide low filling pressures. In addition, some neobladder designs, e.g., the Studer reservoir, use a long isoperistaltic proximal limb, which provides resistance to retrograde flow and therefore additional anti-reflux protection [16]. In this study, reflux was completely prevented by placing pressure on the corresponding point on the abdominal surface while straining for voiding urine in a low pressure reservoir in all patients.

The Studer or Hautmann pouch is widely used these days [17, 18], yet there are still some drawbacks. The afferent limb of the Studer pouch is anastomosed with the bilateral ureters together, so the left one should be tunneled under the mesosigmoid for anastomosis with the afferent ileal segment using the Wallace technique. Left stenosis occurred twice as frequently as on the right side because of extensive dissection [19]; we also had to address both sides although impairment only occurred unilaterally. However, with our technique, we effectively avoid these drawbacks because of the bilateral ureteroileal anastomosis respectively and relatively less ureteral dissection. We had one patient with left ureteroileal anastomotic stenosis 6 months after surgery; however, we only need dealing with the stenosis ipsilaterally after failure of endoscopic stenosis dilation

Herr et al. [20] suggested that pelvis recurrence rates are 10–13% in lymph node metastasis after radical cystectomy. In our study, one patient, age 38, with one metastatic pelvic lymph node had pelvic tumor recurrence 15 months after surgery, which lead to bilateral hydonephrosis 25 months postoperatively. The 10-month interval was because the pelvic recurrence barely affected the anterior pathway of the ureteroileal anastomosis, until it had grown to a larger size; we transformed the diversion into a ureterocutaneoplasty safely after that. The ureteroileal anastomosis can be easily and safely transformed into a ureterocutaneoplasty under local anesthesia because the ureteroileal anastomosis is positioned more superficially than usual.

This new ureteroileal anastomotic technique uses a simple end-to-end method, just like a reflux technique, but functions as an anti-reflux technique. In this study, reflux can be completely prevented by placing pressure on the corresponding point on the abdominal surface while straining for voiding urine in all patients; contracting the abdominal muscle is also helpful.

We spent more operative time during the first case where we performed this technique; but, we rapidly decreased operative time to 360 min with an ideal estimated blood loss and blood transfusion rate. The median postoperative stay was 21 days and no patients died during the perioperative period. Six of seven patients had short-term complications but most were Clavien I which resolved either by themselves or after a short period of indwelling catheterization.

The extraperitoneal approach of radical cystectomy and ileal orthotopic substitution has been shown to result in less blood loss, fewer complications, and rapid recovery [21]. We adopted this approach in order to avoid disturbing the neobladder and anastomotic stoma by intraperitoneal organs.

An ideal orthotopic neobladder should have many characteristics, such as low pressure, 500-ml functional volume, complete continence and voiding, and low complication rates [3, 12, 22]. This modified spheroidal-shaped ileal orthotopic bladder substitution has enough capacity and compliance. No patients developed urinary incontinence, urinary tract infection, urethral stenosis, or metabolic acidosis. Continent diversion imposes an extra metabolic burden upon the kidneys, as urinary excretion products are resorbed through the mucosal surface. Typically, only mild metabolic abnormalities will be seen in patients who have normal renal function. In this study, there was no difference between preoperative and postoperative renal function [3].

There were still several limitations that should be noted. First, it was a retrospective study with inherent flaws. Second, relatively few patients were included in our study and only males were involved. A large series including female patients is required to support the advantages of this technique. Finally, this type of technique is demanding compared with other kinds of techniques; other surgeons are needed to confirm its repeatability before widespread acceptance.

## Conclusions

Herein, we described a feasible technique modification in detail, which provided advantages for effective anti-reflux by mechanical finger pressure and abdominal contraction, a low incidence of stricture, and ease for a second operation. The rate of upper tract preservation was excellent in the long-term follow-up period. There were, however, some shortcomings to this technique and more cases with longer follow-up periods are needed to confirm the outcomes.

## Data Availability

The data of this study are private; the reasons are confidentiality issues and ethical issues.
